# 液相色谱梯度洗脱中的谱带压缩效应

**DOI:** 10.3724/SP.J.1123.2020.07042

**Published:** 2021-01-08

**Authors:** Weiqiang HAO, Lijuan LIU, Qiaoyin SHEN

**Affiliations:** 1.常州工程职业技术学院, 江苏 常州 213164; 1. Changzhou Vocational Institute of Engineering, Changzhou 213164, China; 2.南京大学常州高新技术研究院, 江苏 常州 213164; 2. High-Tech Research Institute of Nanjing University, Changzhou 213164, China

**Keywords:** 液相色谱, 梯度洗脱, 谱带压缩, 溶剂强度模型, liquid chromatography (LC), gradient elution, peak compression, solvent strength model

## Abstract

谱带压缩效应是梯度洗脱区别于等度洗脱的重要特征。经典的范德姆特(van Deemter)理论塔板高度方程基于等度洗脱推导得到,因此不能对谱带压缩效应进行描述。在梯度洗脱中,保留因子(*k*)会随流动相组成(*φ*)的改变而发生变化,这就使得对梯度洗脱机理的研究要比等度洗脱复杂许多。该文对近10年来谱带压缩效应的研究进展,特别是溶剂强度模型(即描述ln *k*与*φ*关系的数学表达式)的非线性特征对谱带压缩因子(*G*)的影响进行了述评,指出为了更好地认识谱带压缩效应需要将这种非线性因素考虑在内。

## Abstract (en)

Peak compression is a unique feature of gradient elution and is non-existent in isocratic elution. Since the classical plate height equation, which is also called as van Deemter equation, is derived by assuming isocratic elution, it cannot be used to account for the effects of peak compression. As opposed to the case of isocratic elution, the retention factor (*k*) varies with the mobile phase composition (*φ*) under gradient elution, thereby complicating mathematical analysis. Herein, the research progress on peak compression in the past decade, especially the effect of the nonlinear feature of solvent strength models (i. e., expressions for ln *k* vs. *φ*), is reviewed. A general expression for the peak compression factor (*G*) is introduced, for which the variation in plate height (*H*) with *φ* is ignored. Based on this equation, the classical equation for *G*, which was first proposed by Poppe and assumes the linear solvent strength model (LSSM) and linear gradient elution, can be derived. The effects of pre-elution of the solute in the initial mobile phase on *G*, which are attributed to the dwelling time of the system, are included in the Poppe equation. When the solvent strength model is nonlinear, e. g., the quadratic solvent strength model (QSSM), the analytical expressions for *G* can also be obtained from the general expression. Under ideal chromatographic conditions, where *H*=0 and the adsorption isotherm is linear, the peak compression is determined by the ratio of the retention factor of the solute in the initial mobile phase to that at the eluted mobile phase composition.

等度洗脱和梯度洗脱是液相色谱的两种基本操作模式。在等度洗脱中,实验参数如流动相组成(即强洗脱溶剂在流动相中的体积分数,*φ*)等为一常数。在梯度洗脱中,*φ*则会随时间(*t*)的改变而发生变化。与等度洗脱相比,梯度洗脱有助于缩短分析时间,减少色谱峰的宽度,在实践中有着广泛应用^[1,2]^。然而溶质的保留因子(*k*)以及传质系数,如溶质沿色谱柱轴向的扩散系数(*D*)以及在固定相和流动相之间的吸附动力学系数(*k*_f_)等,也会随着*φ*的改变而发生变化。在色谱理论中,色谱过程所涉及的对流、扩散、吸附等可通过由偏微分方程组构成的色谱模型进行描述^[3,4]^。物理参数*k*、*D*、*k*_f_等往往出现在偏微分方程的系数之中。对于等度洗脱,这些参数为常数,因此等度洗脱所对应的色谱模型为常系数偏微分方程组。然而对于梯度洗脱,*k*、*D*、*k*_f_则会随着*φ*的改变而发生变化,因此其所对应的色谱模型为变系数偏微分方程组,这就增加了数学分析的难度。

“谱带压缩效应”是梯度洗脱的重要特征。在梯度洗脱中,沿着流速方向,谱带后沿所处的流动相洗脱强度将高于前沿。因此,位于谱带后沿的溶质的迁移速率快于前沿,进而导致谱带压缩。在色谱中,溶质的迁移速率等于*u/*(1*+k*),其中*u*为流动相的线性流速^[3]^。因此,谱带压缩的程度与*k*在谱带区间的分布相关。对于等度洗脱,*k*为常数,因此谱带压缩效应在等度洗脱中并不存在。经典的范德姆特(van Deemter)理论塔板高度方程基于等度洗脱推导得到,因此并未包含谱带压缩效应的影响^[5]^。但是对于梯度洗脱,由范氏方程所描述的因扩散或吸附等造成的谱带展宽现象(即理论塔板高度*H*>0)依然存在。在梯度洗脱中谱带压缩和谱带展宽现象同时并存,这也增加了分离机理研究的难度。Gritti和Guiochon^[6,7]^指出,目前对于谱带压缩效应的研究尚未引起足够的重视。

## 1 Poppe公式

对于最简单的梯度洗脱情形,梯度曲线为线性,


(1) *Φ*(*t*)=*φ*_0_+*Bt*

其中,*Φ*(*t*)为输入系统的流动相组成随时间*t*(min)变化的程序,*φ*_0_为初始流动相组成,*B*为梯度斜率,溶剂强度模型为Snyder等^[8,9]^提出的线性溶剂强度模型(linear solvent strength model, LSSM),


(2) ln *k*=ln *k*_0_-*Sφ*

其中,ln *k*_0_和*S*为溶剂强度参数,Poppe等^[10]^基于平衡-扩散色谱模型(即将导致谱带展宽的所有传质因素集中体现在轴向扩散系数*D*的大小上,通常用符号*D*_a_替代*D*^[3,4]^),推导得到第一个具有严格数学意义,也是在2008年之前唯一一个可用于谱带压缩因子(*G*)计算的数学公式(以下简称Poppe公式)^[6]^,


(3) $$G=\frac{\sigma_{L}}{\sqrt[{}]{\mathrm{HL}}}=\frac{\sqrt[{}]{p^{2}/3+p+1}}{1+p}$$

其中,*σ_L_*为色谱峰沿色谱柱轴向的标准偏差(cm), *H*为理论塔板高度(cm), *L*为色谱柱长(cm),参数*p*通过公式(4)进行计算,


(4) $$p=\frac{k_{\varphi 0}\mathrm{BS}t_{0}}{1+k_{\varphi 0}}$$

其中,*t*_0_为死时间(min), *k_φ_*_0_为溶质在初始流动相中的保留因子。Poppe公式在推导的过程中还遵循以下两个假设^[6,10]^: (1)*H*不随*φ*的改变而发生变化;(2)梯度曲线在迁移过程中未发生变形,即固定相对流动相组成溶剂的吸附可忽略不计。在得到*G*值后,色谱峰宽(*W*)可通过下式进行计算:


(5) $$W=\frac{4Gt_{0}(1+k_{\varphi R})}{\sqrt[{}]{N}}$$

其中,*N*为色谱柱的理论塔板数(*N=L/H*), *φ*_R_为溶质流出色谱柱时所对应的流动相组成,*k_φ_*_R_为*φ*_R_情形中的保留因子。然而,实践中由公式(3)~(5)计算得到的峰宽理论值往往小于实验测定值^[11,12]^。Snyder等^[13]^尝试引入经验参数*J*,即将公式(5)中的*G*替换为*GJ*,从而消除这种偏差。然而,*J*的物理意义至今仍不明确,这在一定程度上限制了人们对于谱带压缩效应的认识。

## 2 Poppe公式的改进

在实践中ln *k*-*φ*曲线往往呈现出非线性的特征^[14,15]^。Carr等^[16]^指出,受制于LSSM的局限,著名色谱软件DryLab将无法准确预测梯度洗脱中的色谱图。为了更为准确地描述ln *k*与*φ*之间的关系,一些学者提出了具有如下形式的拟线性或非线性溶剂强度模型^[17,18,19]^,


(6) ln *k*=ln *k*_0_*-S*_1_*φ*+*S*_2_*φ*^2^

(7) $$\ln k=\ln k_{0}-\frac{S_{1}\varphi }{1+S_{2}\varphi }$$

(8) $$\ln k=\ln k_{0}+2\ln(1+S_{2}\varphi )-\frac{S_{1}\varphi }{1+S_{2}\varphi }$$

其中,ln *k*_0_、*S*_1_、*S*_2_为溶剂强度参数。Neue等^[20]^通过研究指出,为了正确认识梯度洗脱中的谱带压缩效应,需要将ln *k*-*φ*曲线的非线性特征考虑在内。在计算中,Neue等^[20]^将*k_φ_*_R_的实验测定值替换基于LSSM得到的理论值,由此计算得到*W*的理论值与实验值之间的误差可降低至7%。Neue等^[20]^研究工作的不足是尚未将ln *k*-*φ*曲线的非线性特征对*G*值的影响进行校正。

作为对Poppe工作的改进,Gritti和Guiochon^[6,7]^基于非线性溶剂强度模型,以平衡-扩散色谱模型为研究对象,探讨了*G*的通用数学表达式。本文作者则根据传输色谱模型(即将导致谱带展宽的所有传质因素集中体现在吸附动力学系数*k_f_*的大小上,通常用符号*k*_fL_替代*k_f_*^[3,4]^)展开研究,得到了具有如下形式的理论塔板高度方程^[21]^,


(9) $$H_{ap}=\frac{L\sigma_{t}^{2}}{t_{R}^{2}}=\frac{t_{0}{k^{2}}_{\varphi R}}{t_{R}^{2}}\{\frac{H_{\varphi 0}t_{D}(1+k_{\varphi 0}{)}^{2}}{k_{\varphi 0}^{3}}+\int_{0}^{t_{R}-t_{0}-t_{D}}\frac{H_{\Phi (t)}[1+k_{\Phi (t)}{]}^{2}}{k_{\Phi (t)}^{3}}dt\}$$

其中,σt为色谱峰沿时间坐标的标准偏差(min), tR为保留时间(min), tD为流动相从输液系统中的混合器到达色谱柱入口所必需的时间(min), Hap为表观理论塔板高度(cm), k、H中的下标为其所对应的流动相组成。公式(9)与Gritti和Guiochon得到的结果基本一致^[22]^。不同之处在于公式(9)进一步考虑了梯度延迟时间tD对色谱峰宽的影响。在实践中,任何一台HPLC仪都不可避免地会存在梯度延迟。这就意味着在分离的过程中,在梯度曲线追上溶质之前,溶质将会随着初始流动相在色谱柱中进行迁移。溶质在初始流动相中的迁移行为可通过无量纲值Z0/L=tD/(t0kφ0)进行度量,其中Z0为溶质随初始流动相在柱中的迁移距离(cm)^[23]^。由于不同HPLC仪的tD都不一样,研究tD对溶质色谱行为的影响对于分离结果的准确预测,以及分析方法在不同仪器之间的转移将具有重要意义。

当H随φ的变化可忽略不计时,由公式(9)可得到G的一个通用表达式^[23]^,


(10) $$G^{2}=\frac{{k^{2}}_{\varphi R}}{t_{0}(1+k_{\varphi R}{)}^{2}}\{\frac{t_{D}(1+k_{\varphi 0}{)}^{2}}{k_{\varphi 0}^{3}}+\int_{0}^{t_{R}-t_{0}-t_{D}}\frac{[1+k_{\Phi (t)}{]}^{2}}{k_{\Phi (t)}^{3}}dt\}$$

对于Poppe所研究的LSSM-线性梯度洗脱情形,由(10)式可得:


(11) $$G^{2}=\frac{t_{D}}{t_{0}k_{\varphi 0}}\cdot q^{2}+\left(1-\frac{t_{D}}{t_{0}k_{\varphi 0}}\right)\cdot \frac{1+q+q^{2}}{3}=\frac{1+q+q^{2}}{3}+\frac{t_{D}}{t_{0}k_{\varphi 0}}\cdot \frac{\left(2q+1)(q-1\right)}{3}$$

其中,


(12) $$q=\frac{1/k_{\varphi 0}+1}{1/k_{\varphi R}+1}=\frac{1+k_{\varphi 0}}{k_{\varphi 0}/k_{\varphi R}+k_{\varphi 0}}$$

当tD/(t0kφ0)被忽略不计时(Poppe在推导公式(3)时采用了Snyder等^[8,9]^提出的忽略了tD/(t0kφ0)影响的保留时间公式),由公式(11)可得到Poppe公式(这时q=1/(1+p))^[23]^。当ln k-φ曲线的非线性对G的影响被考虑在内时,如通过Schoenmakers等^[17]^提出的二次溶剂强度模型(quadratic solvent strength model, QSSM,即公式(6))描述ln k与φ之间的关系,对于公式(1)描述的线性梯度洗脱,由公式(10)可得^[23]^:


(13) $$G^{2}=\frac{{k^{2}}_{\varphi R}}{t_{0}(1+k_{\varphi R}{)}^{2}}\left\{\frac{t_{D}(1+k_{\varphi 0}{)}^{2}}{k_{\varphi 0}^{3}}+\frac{\sqrt{\pi /S_{2}}}{2Bk_{0}}exp\left(\frac{S_{1}^{2}}{4S_{2}}\right)\cdot \left\{\frac{1}{\sqrt{3}k_{0}^{2}}exp\left(\frac{S_{1}^{2}}{2S_{2}}\right)erf[\sqrt{3}F(\varphi_{0}),\sqrt{3}F(\varphi_{R})]+\right.\right.\left.\left.\frac{\sqrt{2}}{k_{0}}exp\left(\frac{S_{1}^{2}}{4S_{2}}\right)erf[\sqrt{2}F(\varphi_{0}),\sqrt{2}F(\varphi_{R})]+erf[F(\varphi_{0}),F(\varphi_{R})]\right\}\right\}$$

其中,


(14) $$F\left(x\right)=\frac{2S_{2}x-S_{1}}{2\sqrt{S_{2}}}$$

基于公式(13)计算得到的峰宽理论值与实验值更为吻合^[23,24]^。对于Neue等^[20]^研究得到的实验数据,当考虑ln k-φ曲线的非线性,以及H随φ的变化对G的影响时,计算得到的峰宽理论值与实验值之间的误差可由7%降低至2%^[24]^。这一结果表明,以往研究报道的梯度洗脱中峰宽的理论值与实验值之间存在较大偏差(即通过Snyder提出的经验参数J进行衡量),可能更多的是由于计算方法不当所致。

## 3 理想情形中的谱带压缩效应

基于理想色谱模型对梯度洗脱的分离机理展开研究,将有助于认识谱带压缩效应的极限情形^[25]^。在理想色谱模型中,引起谱带展宽的传质动力学因素被忽略不计(即*H*=0)。因此,谱带在迁移的过程中将仅受到谱带压缩效应的影响,压缩程度将最大化。对于分析型液相色谱(即溶质的浓度位于吸附等温线的线性区间),假定进样函数是宽度为*T*_inj_的矩形进样。对于理想情形中的等度洗脱,由于谱带的形状不会随溶质在柱中的迁移而发生改变,因此色谱柱出口处的谱带宽度(*W_G_*)将等于进样宽度*T*_inj_。而在梯度洗脱中,由于存在谱带压缩效应,*W_G_*<*T*_inj_。对于理想情形中的梯度洗脱,定义*G*_ideal_=*T*_inj_/*W_G_*。当*T*_inj_足够小时,可以证明^[24]^


(15) $$G_{ideal}=\frac{T_{inj}}{W_{G}}=\frac{k_{\varphi 0}}{k_{\varphi R}}$$

上式适用于任意形式的溶剂强度模型以及梯度曲线。需要注意,*G*_ideal_(=*k_φ_*_0_/*k_φ_*_R_)出现在了(12)式的分母之中。对于具有其他溶剂强度形式(如QSSM)的线性梯度洗脱,都可以将*G*(*H*>0)表达为*G*_ideal_(*H*=0)的函数^[24]^。

## 4 结论

在实践中除了流动相组成梯度,还存在温度、pH值、离子强度等多种梯度形式。例如,Gritti^[26]^尝试设计了一种新的加热装置,从而实现温度沿色谱柱轴向的梯度变化。与等度洗脱相比,梯度洗脱在增加分离操作灵活性的同时,也增加了理论研究的难度。虽然梯度洗脱的分离机理非常复杂,但是其依然有规律可循。通过对梯度洗脱中的谱带压缩效应展开研究,将有助于深化人们对于梯度洗脱机理的认识,从而为实践中色谱分离条件的选择提供指导。
